# Data harmonization and federated analysis of population-based studies: the BioSHaRE project

**DOI:** 10.1186/1742-7622-10-12

**Published:** 2013-11-21

**Authors:** Dany Doiron, Paul Burton, Yannick Marcon, Amadou Gaye, Bruce H R Wolffenbuttel, Markus Perola, Ronald P Stolk, Luisa Foco, Cosetta Minelli, Melanie Waldenberger, Rolf Holle, Kirsti Kvaløy, Hans L Hillege, Anne-Marie Tassé, Vincent Ferretti, Isabel Fortier

**Affiliations:** 1 Research Institute of the McGill University Health Centre, 2155 Guy, office 458, Montreal, Quebec H3H 2R9, Canada; 2 Public Population Project in Genomics and Society, Montreal, Canada; 3 Ontario Institute for Cancer Research, MaRS Centre, Toronto, Canada; 4 D2K Research Group, School of Social and Community Medicine, University of Bristol, Bristol, UK; 5 Department of Endocrinology, University of Groningen, University Medical Center Groningen, Groningen, The Netherlands; 6 Department of Chronic Disease Prevention, Public Health Genomics Unit, National Institute for Health and Welfare, Helsinki, Finland; 7 Institute for Molecular Medicine, University of Helsinki, Helsinki, Finland; 8 Department of Epidemiology, University Medical Center Groningen, Groningen, The Netherlands; 9 European Academy of Bolzano/Bozen (EURAC), Center for Biomedicine, Bolzano, Italy; 10 Helmholtz Zentrum München - German Research Center for Environmental Health, Neuherberg, Germany; 11 Department of Public Health and General Practice, HUNT Research Center, Norwegian University of Science and Technology, Trondheim, Norway; 12 Department of Cardiology and Epidemiology, University Medical Centre Groningen, Groningen, The Netherlands; 13 Respiratory Epidemiology, Occupational Medicine and Public Health, National Heart and Lung Institute, Imperial College, London, UK

## Abstract

**Abstracts:**

**Background**

Individual-level data pooling of large population-based studies across research centres in international research projects faces many hurdles. The BioSHaRE (Biobank Standardisation and Harmonisation for Research Excellence in the European Union) project aims to address these issues by building a collaborative group of investigators and developing tools for data harmonization, database integration and federated data analyses.

**Methods**

Eight population-based studies in six European countries were recruited to participate in the BioSHaRE project. Through workshops, teleconferences and electronic communications, participating investigators identified a set of 96 variables targeted for harmonization to answer research questions of interest. Using each study’s questionnaires, standard operating procedures, and data dictionaries, harmonization potential was assessed. Whenever harmonization was deemed possible, processing algorithms were developed and implemented in an open-source software infrastructure to transform study-specific data into the target (i.e. harmonized) format. Harmonized datasets located on server in each research centres across Europe were interconnected through a federated database system to perform statistical analysis.

**Results**

Retrospective harmonization led to the generation of common format variables for 73% of matches considered (96 targeted variables across 8 studies). Authenticated investigators can now perform complex statistical analyses of harmonized datasets stored on distributed servers without actually sharing individual-level data using the DataSHIELD method.

**Conclusion**

New Internet-based networking technologies and database management systems are providing the means to support collaborative, multi-center research in an efficient and secure manner. The results from this pilot project show that, given a strong collaborative relationship between participating studies, it is possible to seamlessly co-analyse internationally harmonized research databases while allowing each study to retain full control over individual-level data. We encourage additional collaborative research networks in epidemiology, public health, and the social sciences to make use of the open source tools presented herein.

## Introduction

The benefits of harmonizing and pooling research databases are numerous. Integrating harmonized data from different populations allows achieving sample sizes that could not be obtained with individual studies [1-4], improves the generalizability of results [3-5], helps ensure the validity of comparative research [6,7], encourages more efficient secondary usage of existing data [8], and provides opportunities for collaborative and multi-centre research [9-12]. Governments, funders, and researchers alike have been stressing the importance of harmonization and collaborative use of data and samples in the population health and biobanking fields over the past half-decade [13-21]. However, managing and harmonizing very large amounts of data from different sources is a significant challenge [20,22-24]. Further, ethical, legal, and consent-related restrictions associated with sharing or pooling of individual-level data represent a common dilemma faced by international research projects and networks [25,26]. Web-based networking technologies and new database management systems are at the forefront of providing solutions to some of these dilemmas [27-32]. When combined with strong collaboration between partners, such tools allow us to interconnect distributed databases through database federation systems and assure secure and effective analysis of complex datasets across research centres while retaining individual-level data within host institutions of participating studies.

BioSHaRE (Biobank Standardisation and Harmonisation for Research Excellence in the European Union) is a Seventh Framework Programme (FP7) funded project whose aim is developing data harmonization tools and standardized IT systems for existing biobanks and cohorts across Europe, and apply them to conduct pan-European epidemiological research [33]. As a core project of BioSHaRE, the Healthy Obese Project (HOP) piloted retrospective data harmonization and database federation tools to effectively assess the compatibility of collected data and to safely federate research databases in order to conduct obesity-related research, with a focus on the characterization of metabolically healthy obese individuals [34,35]. Since ‘healthy obesity’ is rather rare, researchers need a large numbers of subjects to explore its determinants and consequences. To investigate subgroups, even larger numbers are needed, making the HOP a good case study for harmonization and co-analysing data from several large population-based studies.

The data harmonization and database federation methodology and infrastructure developed and piloted under BioSHaRE’s HOP is founded on the DataSHaPER (DataSchema and Harmonization Platform for Epidemiological Research) harmonization approach [22,37] and on information technology tools developed by OBiBa (Open Source Software for BioBanks) [38]. These have been recently integrated into a platform to support retrospective harmonization and integration of data [39] by the Maelstrom Research team [40]. The current paper presents the stepwise data harmonization and database federation process employed for the HOP (Table 1) and the information technology tools developed to support it [38]. Resources described in this paper are currently being used by BioSHaRE to harmonize, integrate and jointly analyse data collected by eight population-based cohorts across Europe. Additional studies are joining the project and making use of these tools on a regular basis. The infrastructure described in this paper is helping to create a collaborative environment for BioSHaRE investigators. It aims to facilitate: (1) transforming data collected by existing studies into a common format through the use of processing algorithms; (2) interconnecting harmonized databases located in different countries and institutions across Europe; and (3) achieving combined statistical analyses of these datasets without pooling or sharing individual-level data.

**Table 1:** The Healthy Obese Project data harmonization and database federation step-by-step process

**Step**	**Description**
Study recruitment and documentation	Studies are recruited to participate in the HOP and their key characteristics (e.g. design, sampling frame) are catalogued on the BioSHaRE website (www.bioshare.eu).
Harmonized variable selection and definition	A set of ‘target’ variables required to answer obesity-related research questions is identified at workshops bringing together BioSHaRE investigators.
Study variable identification and harmonization potential assessment	By analysing participating studies’ questionnaires, standard operating procedures, and data dictionaries, the potential for each study to generate this set of target variables is determined. Study-specific variables required to generate target variables are identified.
Data processing	Secure servers are set-up in each study’s host institution and the subsets of data required to generate target variables are loaded onto each of these servers. Processing algorithms transforming study data into the target (i.e. harmonized) format are developed and implemented for each study whenever harmonization is deemed possible.
Harmonized data federation, dissemination and analysis	A password protected web portal federates the servers found in the different study host institutions across Europe and allows remote retrieval of data summaries, descriptive statistics (frequencies, min, max, mean, standard deviation), and contingency tables. For more complex federated data analyses (e.g. linear regressions), the DataSHIELD method [28] is employed in the R software environment [36].

## Study recruitment and documentation

The first step in the data harmonization and database federation process was to recruit studies to participate in the project. To be eligible to participate in the HOP, studies needed to collect comprehensive health outcome, socio-demographic, behavioural, physical and biochemical measures, and allow remote access to aggregated data for statistical analyses. Studies were also required to make study metadata (i.e. questionnaires, data codebooks, standard operating procedures) and ethical and legal documents/policies available to the BioSHaRE coordinating group. A preliminary scan of consents, data access, and IP policies was conducted by the Public Population Project in Genomics and Society (P3G) [41] to assess the potential for each study to participate. Study investigators then submitted formal requests to participate in the project to their respective research ethics or data access committees. Next, key characteristics of participating studies were documented using a standardized online description form found on the Mica-powered BioSHaRE website (see *“What is Mica?”* below) [33]. These characteristics included general study design, number of participants, participant characteristics, methods of recruitment, number and type of biological samples collected, and data and sample access conditions. Cataloguing such information helped in better understanding the level of heterogeneity across study designs as well as potential sample sizes available for analyses. Table 2 lists the eight studies participating in the HOP to date.

**Table 2:** Healthy Obese Project participating studies to date, number of participants, host institutions, and location

**Study name**	**Acronym**	**Number of participants in the HOP**	**Host institution**	**Location**
Cooperative Health Research in South Tyrol Study	CHRIS	1116	European Academy of Bolzano	Bolzano, Italy
KORA Cooperative Health Research in the Region of Augsburg	KORA	18 000	Helmholtz Center Munich	Augsburg, Germany
LifeLines Cohort Study	LifeLines	93 000	University Medical Center Groningen	Groningen, The Netherlands
Microisolates in South Tyrol Study	MICROS	1300	European Academy of Bolzano	Bolzano, Italy
National Child Development Study	NCDS	18 558	University of Leicester	Leicester, United Kingdom
FINRISK 2007 Study	FINRISK 2007	10 000	National Institute for Health and Welfare	Helsinki, Finland
Nord-Trøndelag Health Study	HUNT	78 968	Norwegian University of Science and Technology	Trondheim, Norway
Prevention of REnal and Vascular ENd-stage Disease study	PREVEND	8592	University Medical Centre Groningen	Groningen, The Netherlands

### What is Mica?

Mica [38] is a software application developed to create web portals for individual epidemiological studies or for study consortia. Features supported by Mica include a standardized study catalogue, data dictionary browsers, online data access request forms, and communication tools (e.g. forums, events, news). When used in conjunction with the Opal software, Mica also allows authenticated users to perform distributed queries on the content of study databases hosted on remote servers and retrieve summary statistics and contingency tables.

## Harmonized variable selection and definition

In the second step of the process, HOP investigators convened to select and define a set of ‘target’ variables required to answer specific obesity-related research questions. This set of variables, or DataSchema [22], acted as a template for the retrospective harmonization process by defining the common format measures to be derived using data of participating studies. In order to allow multiple studies to participate in a collaborative endeavour while ensuring validity of the scientific output, the development of a DataSchema requires a balance between uniformity (e.g. exact same question wording and data collection procedures) and acceptance of certain level of heterogeneity across studies (e.g. slightly different wording or procedures). Two workshops (March and June 2012) bringing together BioSHaRE investigators from across Europe and Canada were organized to identify and define target variables making up the HOP DataSchema. Each workshop respectively focused on selecting variables to answer the following research questions: (1) What is the prevalence of obese individuals not showing increased metabolic or cardiovascular risk in each study (i.e. the ‘healthy obese’)?; and (2) What are the lifestyle and behavioural risk factors associated with ‘healthy obesity’? Following the workshops, the DataSchema went through iterative rounds of revisions through teleconferences and electronic communication to arrive at a consensus on target variables (e.g. weight), definitions (e.g. measured weight), and format (e.g. weight in Kg). For certain areas of information, international standards and classifications were used to define target variables and thereby facilitate international comparison of key concepts. For example, education-related DataSchema variables were developed using UNESCO’s International Standard Classification of Education [42], while the ‘current occupation’ variable was developed using the International Labour Organization’s International Standard Classification of Occupations [43]. Once finalized, DataSchema variables were annotated in a designated section of the Mica-powered BioSHaRE website (see https://www.bioshare.eu/content/healthy-obese-project-dataschema). To date, 96 variables including anthropometric and biochemical measures, history of obesity-related disease outcomes, socio-demographic status, and lifestyle and risk factors make up HOP DataSchema. New variables, including constructs covering the physical activity domain, will be added to the DataSchema over the course of the project.

## Study variable identification and harmonization potential assessment

As a third step, using study questionnaires, standard operating procedures, and data dictionaries, harmonization team research assistants identified study-specific data covering DataSchema variables and formally assessed the potential for each study to generate each of these variables (96 variables across 8 studies). This step consisted of comparing the full definition and format of a DataSchema variable to study-specific questions, collection procedures and data formats to determine their compatibility. For example, in order for a given study to generate the ‘weight’ DataSchema variables, this variable needed to be objectively measured by a doctor, nurse or technician rather than self-reported by the participant. Not all studies could generate all of the 96 targeted variables. When assessing the harmonization potential, there were two reasons for which a particular study could *not* generate a specific DataSchema variable: either because the study simply did not collect information on the construct measured by a particular targeted variable or because the information the study collected on this construct was deemed incompatible with the DataSchema variable definition (e.g. self-reported weight). Harmonization potential assessment allowed determining which DataSchema variables could be generated by each study and identifying what study-specific data needed to be extracted from central study data repositories to be used in the remainder of the harmonization exercise. The overall harmonization potential assessment showed that 73% of all matches evaluated (96 DataSchema variables for each of the 8 studies) were considered compatible. Some domains of information proved to be more problematic to harmonize than others. For example, the 30 nutritional habit variables showed a harmonization potential of only 37% for all matches evaluated. On the other hand, the nine variables covering disease history and medication use (i.e. stroke, diabetes, high blood pressure, myocardial infarction) were considered compatible with DataSchema formats 97% of the time.

### Data processing

**Figure 1:**
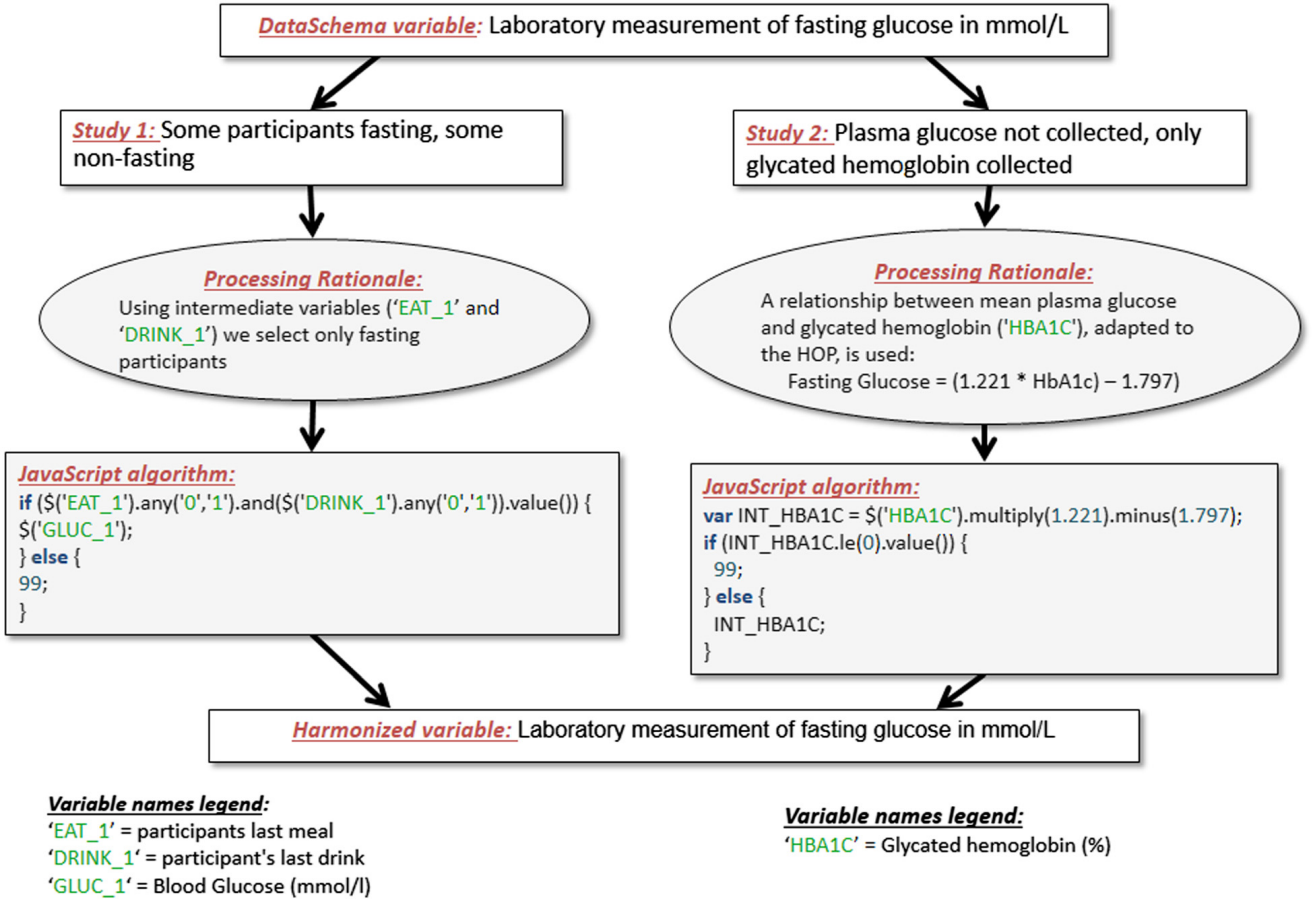
Example of data processing to obtain a common format: deriving the harmonized Fasting Glucose DataSchema variable for two studies.

The fourth step involved processing study-specific data under the DataSchema variable format. This was done with the help of OBiBa’s Opal software (see “*What is Opal?”* below), which was installed on secure servers within the respective host institutions of participating studies (see Table 1). Data dictionaries (i.e. codebooks) of each participating study were converted into a standardized format readable by Opal and loaded onto the server. Each study then extracted data required to generate DataSchema variables (identified in the previous step) from their main database and loaded it on their respective Opal servers. To guide data processing, the reference DataSchema structure (i.e. common variable names, labels, and coding for categories) was also loaded onto each study-specific Opal instance. By accessing aggregate data via remote connections to each study server, data processing was then centrally conducted by the harmonization team to transform study-specific data into the common format defined by the DataSchema. For each DataSchema-variable-to-study match, the rationale describing the procedure to generate the DataSchema variable was first established. This ‘processing rationale’ varied in nature and scope depending on the variable to be harmonized. For example, in some instances, simple recoding of study data categories was sufficient to generate a DataSchema variable in the appropriate format. In other situations, such as for the generation of the harmonized Fasting Glucose variable (Figure 1), data processing had to be supported by a more detailed explanation, which was documented in Opal. Once the ‘processing rationale’ was established, study specific processing algorithms were developed, documented and implemented in Opal, putting to use the software’s ability to compute custom JavaScript code [44] to derive variables. Once executed on study data, algorithms were validated by comparing the distribution and counts of harmonized datasets to the data originally collected by each study. The data processing step ultimately resulted in the creation of one harmonized dataset per participating study, hosted on each host institution’s firewall-protected server.

### What is Opal?

Opal [38] is an software application used to manage study data and includes a software infrastructure enabling data harmonization and data integration across studies. As such, Opal supports the development and implementation of processing algorithms required to transform study-specific data into a common harmonized format. Moreover, when connected to a Mica-web interface, Opal allows users to seamlessly and securely search distributed datasets across several Opal instances.

## Harmonized data federation, dissemination and analysis

The fifth and last step in the process aimed to co-analyse harmonized datasets while addressing ethical and legal restrictions associated with pooling individual-level data. To achieve this, the Opal and Mica software applications were used in parallel to create a federated infrastructure that allows researchers to jointly analyse harmonized data while retaining individual-level data within their respective host institutions. Hence, once harmonized datasets were generated on local Opal servers in each host institution, these servers were securely connected via encrypted remote connections (using HTTPS).

**Figure 2:**
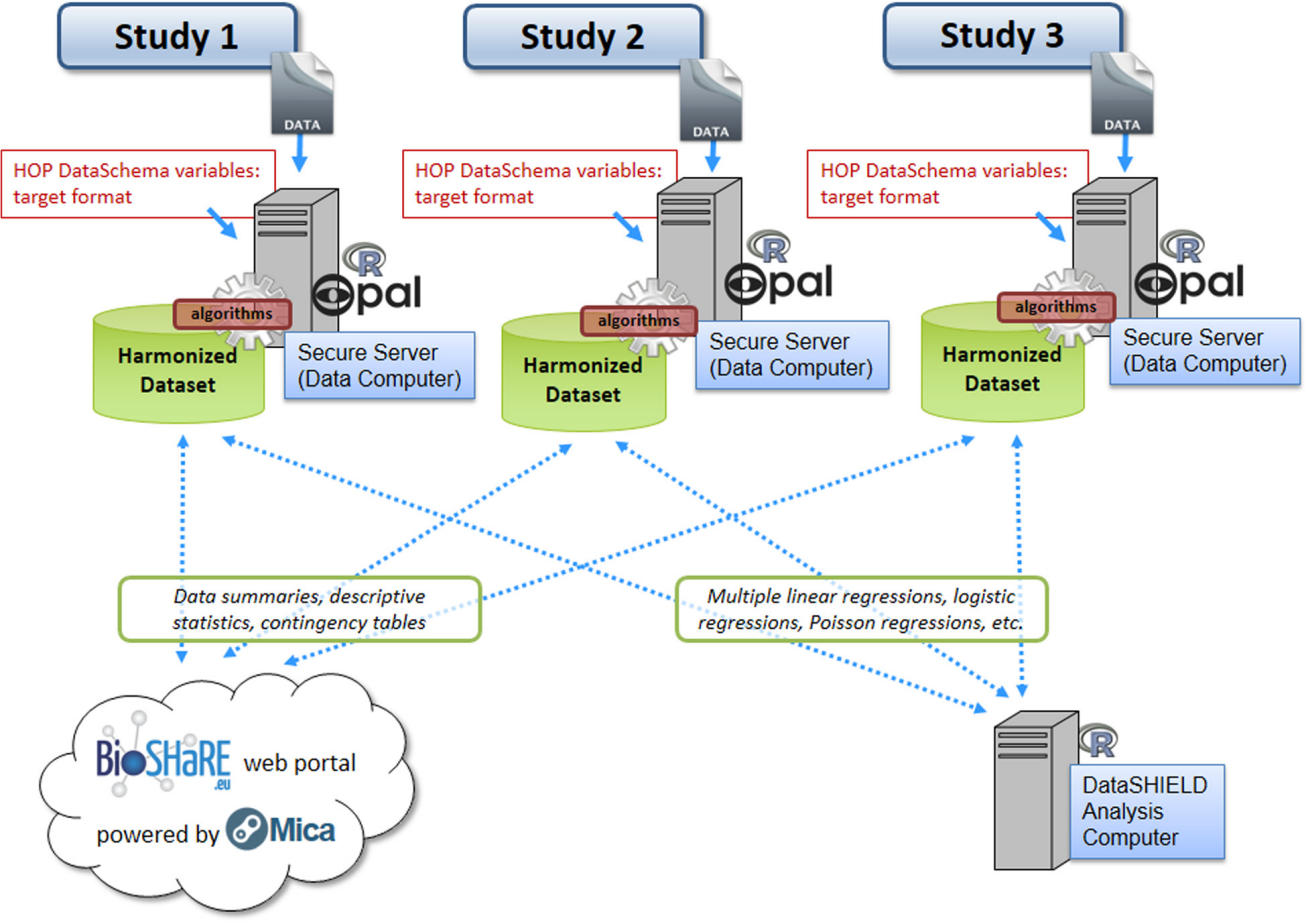
Data harmonization and federated infrastructure for three HOP studies.

Two types of analyses are made available through this framework (see Figure 2). Firstly, once logged on to a password protected section of the Mica-based BioSHaRE.eu website, investigators can securely execute queries allowing them to retrieve data summaries, descriptive statistics (frequencies, min, max, mean, standard deviation), or contingency tables of the harmonized databases hosted on each of the geographically-dispersed Opal servers. Multiple investigators can run such distributed queries simultaneously and in real time on the different Opal servers. Secondly, and to support more complex federated data analyses such as multiple linear regressions, logistic regressions, Poisson regressions, or for undertaking a simple analysis such as executing a *t*-test, the Opal-Mica framework is fully compatible with the DataSHIELD method (see “*What is DataSHIELD*?” below) [28,45]. When a joint analysis is to be undertaken using data from several sources, statistical efficiency and flexibility is often best served by working directly with individual-level data rather than by meta-analysing summarised results from each study [46]. However, important ethico-legal constraints, intellectual property considerations, and/or the physical size of the data to be analysed, often prevent or delay the sharing of individual-level data [47]. Based on parallelized analysis and modern distributed computing, DataSHIELD enables the analysis of harmonized individual-level data without the need to physically pool them [28,45].

### What is DataSHIELD?

DataSHIELD (www.datashield.org) acts as an interface module between the Opal software application and the R software environment [36]. Under DataSHIELD, a central analysis computer (i.e. the computer from which analysis is carried out) coordinates a parallelized simultaneous analysis of the individual-level data on all the data computers (i.e. the secure servers where the individual-level data are stored) by sending blocks of code, in the form of simple analytic commands, to each data computer. These request each server to undertake a particular analysis and to return non-disclosive summary statistics to the analysis computer, that is data which cannot possibly lead to the identification of the individuals to which they relate. For analyses such as the fitting of a generalized linear model, DataSHIELD works iteratively. After each iteration, summary statistics (typically the score vector and information matrix) are returned by each data computer to the analysis computer and the estimates of the model are refined; the process ends when the estimates converge. This enables global updating of the estimated model parameters taking full account of the data from *all* studies simultaneously. In this way, it is possible to fit a mathematical model as if the individual-level data from all studies were pooled centrally on the analysis computer while - in reality – the data never leave their studies of origin, and all that *does* leave are the non-disclosive summary statistics.

### IT requirement for DataSHIELD

The DataSHIELD approach places very few demands on the IT equipment required (Figure 2). The analysis computer can be a standard laptop or desktop running any R console [36] or a rich client such as RStudio [48] with DataSHIELD R packages. The data servers must each be running Opal and R. Using this framework, each Opal instance receives, controls and forwards requests from R running on the analysis computer to R running on the server. The controlled and secured web-based links between the analysis computer and the data computers do not need to carry heavy traffic, and DataSHIELD therefore demands no more than a standard wireless link to a broadband access point. It is also possible to channel communications through study firewall configurations to allow only for analyses from computers at specific IP addresses.

## Conclusion

New Internet-based networking technologies and database management systems are providing the means to support collaborative, multi-centre research in an efficient and secure manner [27-32]. Since its inception in 2010, the BioSHaRE project works at harnessing such resources along with international expertise in order to facilitate cross-border collaborations in the biomedical sciences. The Healthy Obese Project has successfully served to pilot a suite of tools which facilitates: (1) transforming existing data collected by different studies into a common format through the use of processing algorithms; (2) interconnecting harmonized databases located across Europe via a federated web-based infrastructure; and (3) achieving joint statistical analyses of harmonized datasets without pooling or sharing individual-level data.

It must be noted that the data harmonization and database federation work conducted within the BioSHaRE project has required a high level of collaboration between different parties. Active involvement of study investigators, research centre staff, and the BioSHaRE coordinating group was pivotal for the software and information technologies to be of use. Though this initiative has proven to require a high level of coordination, the infrastructure that results from it has a number of strengths. First, using the Mica-Opal federated framework, studies retain all control over individual-level data since local Opal instances compute aggregate data before sending results to the central Mica web portal, or to the analysis computer running the DataSHIELD R packages. Since either Mica or the analysis computer act as brokers to securely fetch information from each Opal instance, investigators querying data therefore never connect directly to the servers hosting individual-level data. Secondly, once harmonized datasets are derived on each participating study’s server, they can be used and reused for multiple collaborative research projects. Third, allowing investigators to safely and remotely analyse data (i.e. produce summary statistics, contingency tables, logistic regressions) at their convenience and in real time limits the burden associated with filing multiple data access requests at multiple research centres, thereby saving principal investigators and study managers time and resources. Lastly, Opal-Mica federated infrastructure features such as encrypted remote connections (using HTTPS), user authentication, and control over user access and permissions (e.g. dataset visibility, import/export, data manipulation) effectively ensures that participant data privacy and confidentiality are respected across studies in a collaborative research context.

The HOP pilot project is helping to optimize the tools and methods presented herein and to add new data analysis features to these tools in the aim of constructing a more robust, efficient, scalable and automated framework to support secure analysis of harmonized data in BioSHaRE and other collaborative projects. Through this pilot project, we have shown that seamlessly and securely co-analysing internationally harmonized research databases is possible. We hope that the open source tools presented in this paper will be of interest to additional research networks in epidemiology, public health, and the social sciences in the future. Opal and Mica software as well as the DataSHIELD R packages are freely available to the research community under the GPL3 license at https://www.obiba.org.

## Competing interests

The authors declare that they have no competing interests.

## Authors’ contributions

DD, VF, PB, YM, AG, IF contributed to the conception, design and drafting of the manuscript. BW, MP, RS, AM contributed to coordination of the Healthy Obese Project and to the drafting of the manuscript. LF, CM, MW, RH, KK, HH contributed to the acquisition and interpretation of the study-specific data and to the drafting of the manuscript. All authors read and approved the final manuscript.
